# Maternal Diabetes Deregulates the Expression of Mecp2 via miR-26b-5p in Mouse Embryonic Neural Stem Cells

**DOI:** 10.3390/cells12111516

**Published:** 2023-05-30

**Authors:** Sukanya Shyamasundar, Seshadri Ramya, Deepika Kandilya, Dinesh Kumar Srinivasan, Boon Huat Bay, Suraiya Anjum Ansari, S Thameem Dheen

**Affiliations:** 1 Department of Anatomy, Yong Loo Lin School of Medicine, National University of Singapore, Singapore 117594, Singapore; 2 Department of Biochemistry and Molecular Biology, College of Medicine and Health Sciences, United Arab Emirates University, Al Ain P.O. Box 15551, United Arab Emirates

**Keywords:** maternal hyperglycemia, microRNA, synapse, neurodevelopmental disorders

## Abstract

Maternal diabetes has been associated with a greater risk of neurodevelopmental disorders in offspring. It has been established that hyperglycemia alters the expression of genes and microRNAs (miRNAs) regulating the fate of neural stem cells (NSCs) during brain development. In this study, the expression of methyl-CpG-binding protein-2 (Mecp2), a global chromatin organizer and a crucial regulator of synaptic proteins, was analyzed in NSCs obtained from the forebrain of embryos of diabetic mice. Mecp2 was significantly downregulated in NSCs derived from embryos of diabetic mice when compared to controls. miRNA target prediction revealed that the miR-26 family could regulate the expression of Mecp2, and further validation confirmed that Mecp2 is a target of miR-26b-5p. Knockdown of Mecp2 or overexpression of miR-26b-5p altered the expression of tau protein and other synaptic proteins, suggesting that miR-26b-5p alters neurite outgrowth and synaptogenesis via Mecp2. This study revealed that maternal diabetes upregulates the expression of miR-26b-5p in NSCs, resulting in downregulation of its target, Mecp2, which in turn perturbs neurite outgrowth and expression of synaptic proteins. Overall, hyperglycemia dysregulates synaptogenesis that may manifest as neurodevelopmental disorders in offspring from diabetic pregnancy.

## 1. Introduction

Diabetes during pregnancy and the increased risk of neurodevelopmental disorders in offspring have been well established through recent meta-analyses [1,2,3,4]. The offspring of diabetic mothers show poor cognitive functions [5], language [6] and developmental delays [7], and thus it is crucial to understand the molecular basis of maternal diabetes-induced neurodevelopmental deficits. The development and function of the nervous system are regulated by genetic and epigenetic mechanisms, including DNA methylation. Methyl-CpG binding domain (MBD) containing proteins dynamically regulate gene expression and function in the brain [8], by binding to methylated DNA. Methyl-CpG-binding protein-2 (Mecp2), a multi-functional protein that is implicated in several neurodevelopmental disorders, including Rett syndrome, a type of autism spectrum disorder (ASD) [9,10], was the first MBD to be identified. Further, Mecp2 plays a significant role during neurogenesis and synaptogenesis by regulating a wide network of neurodevelopmental genes and microRNAs (miRNAs) [11,12], as well as mediating other epigenetic mechanisms [13,14]. 

Mecp2 has been shown to determine dendritic morphology and synaptic plasticity. Further, loss or gain of function of Mecp2 is associated with neurological and behavioral deficits [15], suggesting that precise levels of Mecp2 are crucial for proper brain wiring and neuronal connectivity. The spatio-temporal expression pattern of Mecp2 has been attributed to the regulatory potential of its 3’UTR. The 3’UTR is well conserved and extraordinarily long, comprising 10.2kb, and contains binding site for several miRNAs, suggesting that miRNAs fine-tune Mecp2 expression. To date, only seven miRNAs have been shown to regulate Mecp2 [16].

miRNAs have diverse functions in brain development and diseases. The cell-type-specific, time-specific and developmental-stage-specific expression of miRNAs regulate the expression of crucial neurodevelopmental genes and biological pathways. Of the known miRNAs, 70% are reported to be ubiquitously expressed in all layers of the brain, emphasizing their role in the central nervous system [17]. We have previously shown that maternal diabetes alters the global miRNA expression profile in mouse embryonic neural stem cells (NSCs) [18]. In this study, the miRNA-26 family (namely, miR-26a-5p and miR-26b-5p) was selected for further analysis as it was predicted to target Mecp2 by bioinformatics analysis. Further, the miR-26 family showed increased expression in NSCs from embryos of diabetic pregnancy (fold change = 1.4 to 1.6) and is implicated in proliferation [19], neurogenesis [20,21] and neuronal differentiation [21,22] during brain development.

We hypothesize that maternal hyperglycemia results in dysregulation of miRNAs, miR-26a-5p and miR-26b-5p in NSCs, which alter the expression of proteins involved in brain development. In vitro culture of NSCs obtained from the forebrain of embryos from control or diabetic pregnant mice was performed [18]. Further validation revealed an increase in the expression of miR-26b-5p and decreased expression of Mecp2 in mouse embryonic NSCs from diabetic pregnancy. Overexpression of miR-26b-5p or knockdown of miR-26b-5p in NSCs altered the expression of Mecp2, suggesting that miR-26b-5p regulates Mecp2. Moreover, miR-26b-5p overexpression or Mecp2 knockdown in NSCs altered neurite outgrowth and synaptogenesis in differentiated cells, suggesting that miR-26b-5p regulated synaptogenesis via Mecp2. Overall, our results suggest that maternal diabetes alters neurite outgrowth and synaptogenesis via deregulation of the miR-26b-Mecp2 pathway, which may form the basis for structural and functional defects in developing brains from the offspring of diabetic pregnant mice.

## 2. Materials and Methods

### 2.1. Animals

The animal procedure was performed as previously described [18,23]. The procedures pertaining to animal usage were approved by the Institutional Animal Care and Use Committee, National University of Singapore (Protocol Numbers: R14-0154, R15-0030). 

### 2.2. In Vitro Culture of NSCs

The culture of NSCs from the forebrain of E13.5 mouse embryos was carried out as previously described [18,23]. Briefly, the forebrain was dissected out from embryos and dissociated. The dissociated cells were washed with PBS and seeded in T-75 flasks (Greiner, Kremsmunster, Austria) containing a 1:1 ratio of DMEM and F12 media (Thermo Fisher Scientific, Waltham, MA, USA) with 10 mM/L D-glucose and other supplements. The flasks were maintained at 37 °C and 5% CO_2_ for 3 to 4 days, after which the neurospheres formed were dissociated and re-plated. The cells were used for different experiments following two passages.

### 2.3. Differentiation of NSCs 

Neurospheres were harvested, trypsinized using TryPLE Select and resuspended in DMEM/F12 medium containing all supplements (listed in Section 2.2) except the growth factor, EGF. FBS (at a final concentration of 2%) was added to the media to induce differentiation of NSCs. The trypsinized cells were cultured in differentiating media at 37 °C and 5% CO_2_ for 72 h before using them to observe the expression of synaptic proteins. For immunofluorescence analysis, the dissociated neurospheres were seeded on 24-well plates containing ornithine-coated coverslips and incubated for 72 h. 

### 2.4. Total RNA Extraction 

The Qiagen miRNEASY kit (Qiagen GmbH, Hilden, Germany) was used to extract total RNA. The extracted RNA was quantified and used for mRNA or miRNA analysis.

### 2.5. cDNA Synthesis and mRNA Analysis

The cDNA synthesis and gene expression analysis were performed as described previously [18,23]. The delta-delta Ct method was used to determine the fold change in gene expression [24]. The primers used and their sequences are detailed in Table 1. 

### 2.6. cDNA Synthesis and miRNA Analysis

cDNA was synthesized using the Universal cDNA synthesis kit II (Exiqon, Woburn, MA, USA). The expression levels of miRNA-26a or miR-26b-5p were determined using specific primers (primer sequences are listed in Table 2) and ExiLENT SYBR Green Master Mix (Exiqon) by qRT-PCR (7900 HT, Applied Biosystems). We used the U6 snRNA (Exiqon) as the internal control. The fold change in miRNA expression was calculated by the delta-delta Ct method.

### 2.7. Western Blot

Total protein was isolated from NSCs using M-PER extraction reagent (Thermo Fisher Scientific, Waltham, MA, USA) and quantitated. A 30µg amount of denatured protein was resolved by SDS-PAGE and transferred to PVDF membranes, and non-specific binding was reduced by blocking in 5% non-fat milk. Primary antibodies rabbit anti-Mecp2 (1:1000, ab2829, Abcam, Cambridge, UK), rabbit anti-PSD-95 (1:1000, ab18258, Abcam, Cambridge, UK), rabbit anti-Synaptophysin (1:1000, ab32127, Abcam, Cambridge, UK), mouse anti-tau (1:1000, Tau46 #4019, Cell Signaling, Danvers, MA, USA), mouse anti-Clathrin1HC (1:1000, SC12734, Santa Cruz, TX, USA) or mouse anti-beta actin (1:5000, A1978, Sigma-Aldrich, St. Louis, MO, USA) was added to the PVDF membrane overnight at 4 °C. The next day, HRP-conjugated anti-mouse or anti-rabbit secondary antibodies (1:1000, anti-mouse HRP-31430; anti-rabbit HRP-31460, Thermo Fisher Scientific) were added. Chemiluminescence signals were captured on X-ray films, and the bands were quantified (GS-800 densitometer, Bio-Rad, Hercules, CA, USA). 

### 2.8. Transfection of miRNA and siRNA

NSCs obtained from embryos of control mice were trypsinized and seeded in 6-well plates (1 × 10^6^ cells/well) in Opti-MEM. The cells were transfected with SiGenome SMARTpool Mecp2 (M-044034-01-0005, Dharmacon, Lafayette, CO, USA) or SiGenome Control pool (Table 3, D-001206-13-05, Dharmacon) using DharmaFECT1 (Dharmacon) for Mecp2 knockdown. 

A 10 nM amount of miR-26b-5p mimics (Ambion, Thermo Fisher Scientific) or inhibitors of miR-26b-5p and the appropriate negative control probes (Table 4, Ambion, Thermo Fisher Scientific) were transfected in NSCs using lipofectamine RNAiMAX (Thermo Fisher Scientific). siRNA-lipofectamine complexes in opti-MEM medium were changed to NSC culture media or differentiation media 6h post-transfection. RNA extraction was performed 48 h post-transfection while protein was extracted 72 h post-transfection.

### 2.9. Immunofluorescence Analysis 

Neurospheres or differentiated NSCs were seeded in poly-L-lysine or poly–L-ornithine-coated coverslips respectively. The cells were fixed with ice-cold 4% paraformaldehyde (PF) and permeabilized with 0.1% Triton-X 100 in PBS. Subsequently, 3% normal goat serum was added to block non-specific binding before incubating with rabbit anti-Mecp2 (1:500, ab2829, Abcam, Cambridge, UK), rabbit anti-PSD-95 (1:500, ab18258, Abcam, Cambridge, UK), rabbit anti-Synaptophysin (1:500, ab32127, Abcam, Cambridge, UK) or mouse anti-tau (1:500, Tau46 #4019, Cell Signaling, MA, USA) overnight at 4 °C. Subsequently, cells were washed and incubated with an anti-mouse or anti-rabbit IgG antibody tagged with a fluorophore (1:100, Chemicon, Temecula, CA, USA) for 1h at room temperature. DAPI (1:5000, D-1306, Sigma, MI, USA) was used to counterstain the nucleus. Finally, fluorescent mounting medium (DAKO, Santa Clara, CA, USA) was used to mount the coverslips onto the glass slides and the images were captured on a confocal microscope (Olympus FV1000).

### 2.10. Neurite Outgrowth Assay

Neurite outgrowth assay was carried out in NSCs transfected with miR-26b-5p mimics or negative control using the neurite outgrowth assay kit (Chemicon, NS220) according to the manufacturer’s instructions. Cell culture inserts (pre-coated with laminin) containing a permeable membrane with pores that are 3 μm wide were used to culture the transfected NSCs in differentiation media. After 48 h of differentiation, the inserts containing the cell bodies on the top surface and the neurites on the lower surface were fixed and washed. Neurite staining solution in the kit was used to stain the neurite, following which the cell bodies were removed using wet cotton swabs before imaging. The images were taken using an SMZ1500 Zoom Stereomicroscope (Nikon). The stain extraction solution was used to extract the stain from inserts and was collected in a 96-well plate. Spectrophotometer Tecan (Infinite F200 PRO) was used to quantify the neurites, and the absorbance was read at 595 nm.

### 2.11. Statistical Analysis

The data are represented as mean ± SD from 3–5 independent experiments. Student’s t-test was performed, and data were considered significant when *p* ≤ 0.05. 

## 3. Results

### 3.1. Maternal Diabetes Downregulates the Expression of Mecp2 in Mouse Embryonic NSCs 

**Figure 1:**
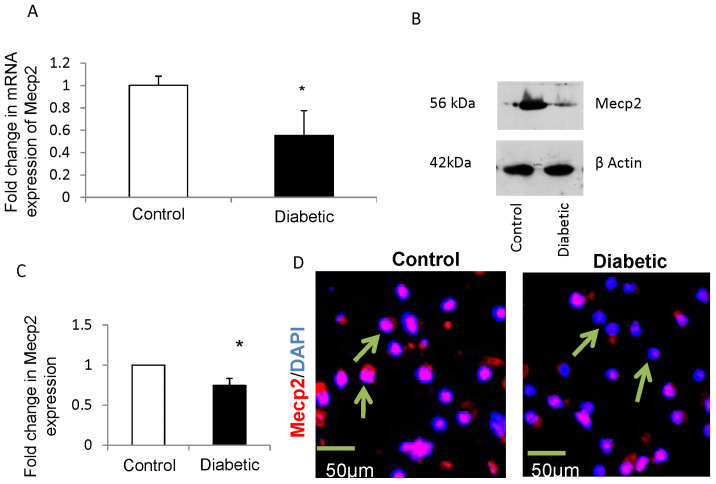
(**A**) qRT-PCR analysis revealed decreased expression of Mecp2 gene in NSCs from embryos of diabetic mice (closed bars) when compared to controls (open bars). n = 4, * *p* < 0.05. (**B**) Representative blot showing decreased expression of Mecp2 protein in NSCs from embryos of diabetic pregnancy when compared with controls. β actin was used as an internal control. (**C**) Quantification of bands by densitometry shows significant downregulation of Mecp2 protein in NSCs from embryos of diabetic mice (closed bar) when compared to controls (open bar). n = 4, * *p* < 0.05. (**D**) Representative confocal images show decreased expression of Mecp2 protein (arrows) in NSCs from embryos of diabetic pregnancy (**right panel**) when compared with controls (**left panel**). Nuclei are stained blue with DAPI. Scale bar, 50 μm.

Firstly, the mRNA and protein expression levels of Mecp2 were quantified in NSCs from embryos of control and diabetic pregnancies by performing quantitative RT-PCR and Western blot, respectively. There was a significant decrease in the expression of Mecp2 gene (Figure 1A) and protein (Figure 1B,C) in NSCs from embryos of diabetic mice when compared to controls. In addition, immunofluorescence analysis confirmed the reduction in the expression of Mecp2 (arrows) in NSCs from embryos of diabetic mice when compared to controls (Figure 1D).

### 3.2. Maternal Diabetes Alters the Expression of miR-26 Family in NSCs from Embryos of Diabetic Pregnancy

**Figure 2:**
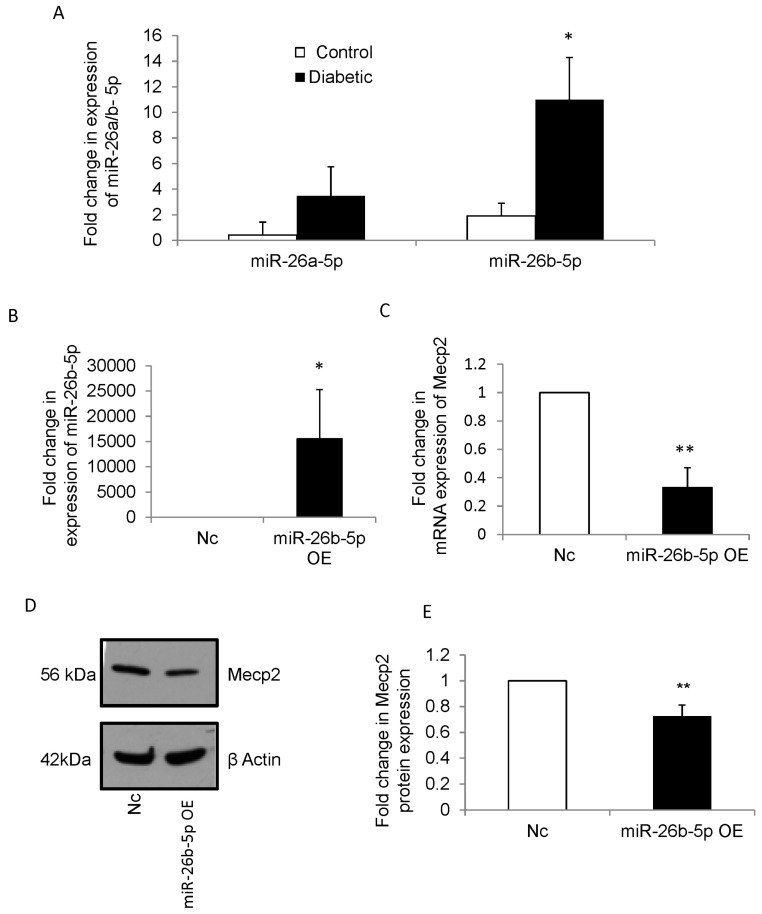

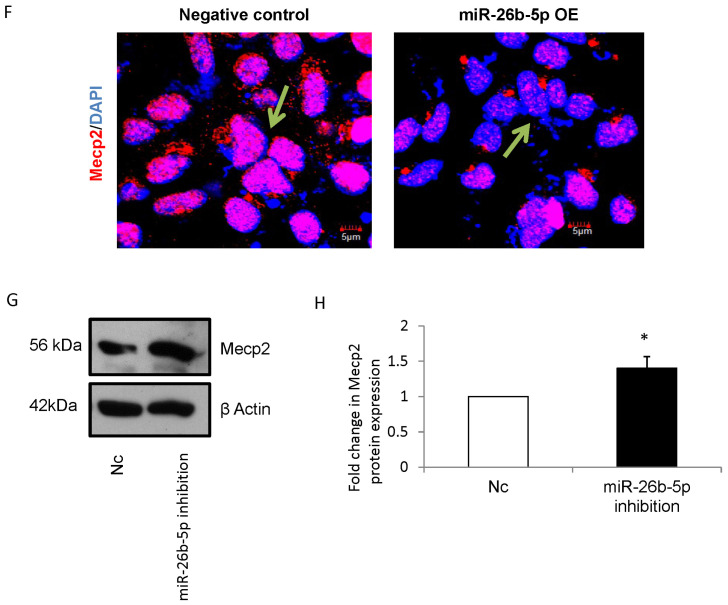
(**A**) qRT-PCR analysis shows upregulation of miR-26 family in NSCs from embryos of diabetic pregnancy compared to that of controls. There is a significant upregulation of miR-26b-5p in NSCs from embryos of diabetic pregnancy (closed bars) when compared to that of control pregnancy (open bars). However, the upregulation of miR-26a-5p expression in NSCs from diabetic pregnancy was not statistically significant, when compared to that of control pregnancy. n ≥ 3, student t-test, * *p* < 0.05. (**B**) qRT-PCR analysis shows >1000-fold upregulation of miR-26b-5p following miR-26b-5p overexpression. n = 4, student t-test, * *p* = 0.05. (**C**) qRT-PCR shows downregulation of *Mecp2* gene following miR-26b-5p overexpression in NSCs from embryos of control pregnancy. n = 4, student t-test, ** *p* < 0.01. (**D**) Representative blot shows decrease in Mecp2 protein expression following miR-26b-5p overexpression in NSCs from embryos of control pregnancy. β actin was used as an internal control. (**E**) Densitometry analysis shows significant decrease in Mecp2 protein expression following miR-26b-5p overexpression (closed bar) when compared to negative control (open bar). n = 4, student t-test, ** *p* < 0.01. (**F**) miR-26b-5p mimics were used to overexpress miR-26b in NSCs from embryos of control pregnancy. Confocal images show decrease in Mecp2 protein (arrows) following miR-26b-5p overexpression (**right panel**), when compared to that of negative control (**left panel**). Nuclei are stained with DAPI. miR-26b-5p OE: miR-26b-5p overexpression. Scale bar, 5 μm. (**G**) Representative blot shows increase in Mecp2 protein expression following miR-26b-5p inhibition in NSCs from embryos of control pregnancy, when compared to that of negative control. β actin was used as an internal control. (**H**) Densitometry analysis shows significant increase in Mecp2 protein expression (closed bar) following miR-26b-5p inhibition in NSCs from embryos of control pregnancy, when compared to that of negative control (open bar). n = 4, * *p* < 0.05. Nc—Negative control, miR-26b-5p OE—Overexpression of miR-26b-5p.

We have previously shown that hyperglycemia deregulates miRNA expression in mouse embryonic NSCs. From the 104 differentially expressed miRNAs [18], miRNA-26 family (miR-26a-5p and miR-26b-5p) was selected for further analysis, and Mecp2 was predicted by TargetScan (mouse) software (version 7.1) to be one of the putative targets of miR-26 family (Supplementary Figure S1A). Quantitative RT-PCR was carried out to validate the expression of miR-26a-5p and miR-26b-5p in NSCs from embryos of control and diabetic pregnancies. There was a significant increase in the expression of miR-26b-5p (but not miR-26a-5p) in NSCs from diabetic pregnancy when compared with controls (Figure 2A). 

### 3.3. miR-26b-5p Regulates Mecp2 in NSCs

To estimate the role of miR-26b in NSCs, loss- and gain-of-function analysis using LNA-modified miR-26b-5p inhibitors or mimics were carried out respectively. miR-26b-5p was overexpressed in NSCs using miR-26b-5p mimics and the expression was quantified by qRT-PCR (Figure 2B). Further analysis showed that miR-26b-5p overexpression resulted in a significant downregulation in both mRNA (Figure 2C) and protein levels of Mecp2 (Figure 2D,E). Immunofluorescence analysis further confirmed the decrease in Mecp2 protein expression following overexpression of miR-26b-5p in NSCs (Figure 2F). On the other hand, inhibition of miR-26b-5p in NSCs resulted in a significant upregulation of Mecp2 protein (Figure 2G,H). Taken together, the results suggest that Mecp2 is a target of miR-26b-5p.

### 3.4. miR-26b-5p Perturbs Synaptic Milieu via Mecp2 in NSCs 

The formation of synapses is crucial for the establishment of neuronal network and brain circuitry [25]. The miR-26 family has been found to regulate neurite outgrowth and the synaptic plasticity [26], while Mecp2 expression levels have been shown to influence the structure of dendrites and number of synapses [27,28,29,30]. Thus, we explored the role of miR-26b-5p and its target gene Mecp2 in neurite outgrowth (the first step during the formation of axons and dendrites) and synaptogenesis (by examining the expression of specific synaptic markers). 

**Figure 3:**
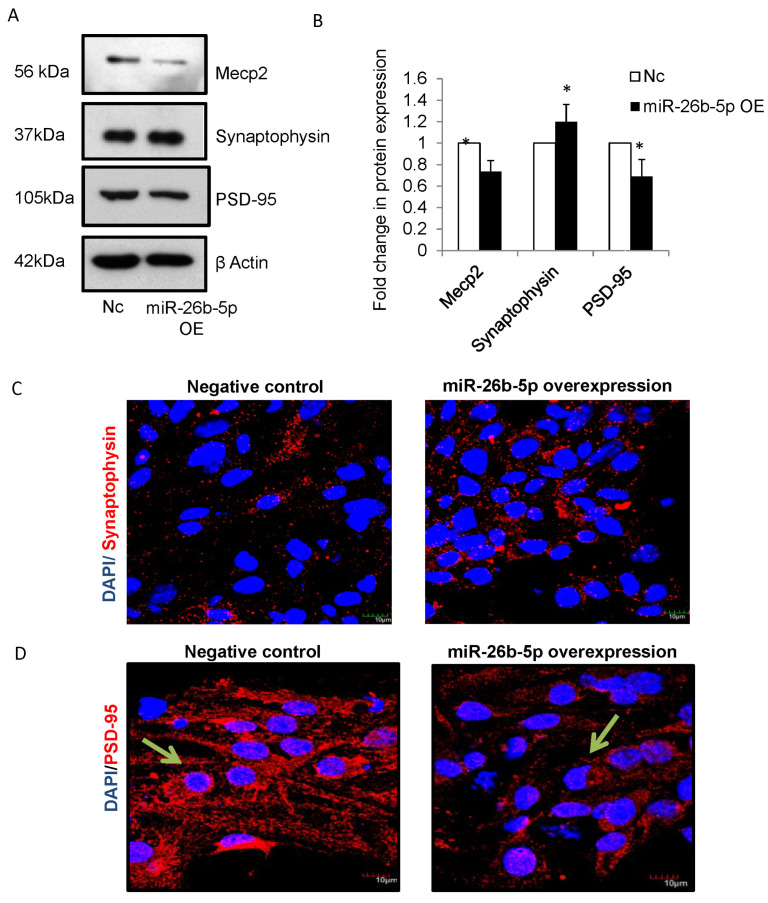

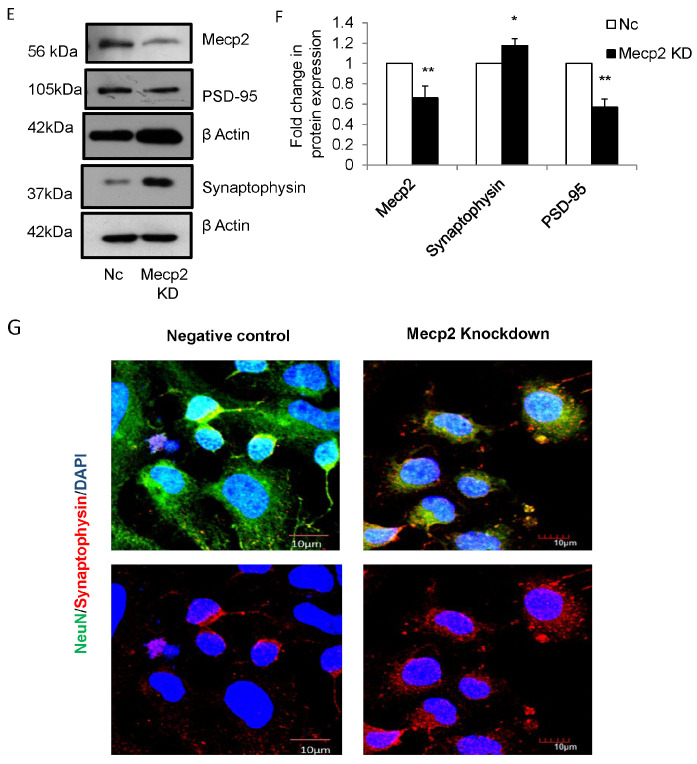
(**A**) Representative blots showing the expression of Mecp2, PSD-95 and Synaptophysin in NSCs following miR-26b-5p overexpression. β actin was used as an internal control. (**B**) Densitometry analysis shows significant downregulation in Mecp2 and PSD-95 proteins, and upregulation in Synaptophysin protein in NSCs following miR-26b-5p overexpression (closed bars) compared to that of negative control (open bars). Nc—Negative control, miR-26b OE—Overexpression of miR-26b-5p. n = 3, * *p* < 0.05. miR-26b-5p was overexpressed in NSCs from embryos of control pregnancy, and immunoexpression of Synaptophysin and PSD-95 was observed using confocal microscopy. (**C**,**D**) Confocal images show an increase in expression of Synaptophysin (**C**) and a decrease in expression of PSD-95 (**D**) in NSCs following miR-26b-5p overexpression (**right panel**) when compared to that of negative control (**left panel**). Scale bar, 10 μm. Nuclei are stained with DAPI. (**E**) Representative blots showing expression of Mecp2, PSD-95 and Synaptophysin in NSCs following Mecp2 knockdown. β actin was used as an internal control. (**F**) Densitometry analysis shows significant downregulation of Mecp2 and PSD-95 protein expression and upregulation of Synaptophysin protein expression in NSCs following Mecp2 knockdown. Nc—Negative control, Mecp2 KD—Knockdown of Mecp2. n = 4, ** *p* < 0.01, * *p* < 0.05. (**G**) Confocal images show an increase in Synaptophysin in NSCs following Mecp2 knockdown (**right panels**) when compared to that of negative control (**left panels**). Nuclei are stained with DAPI. Scale bar, 10 μm.

Firstly, we analyzed the effects of miR-26b-5p overexpression on the expression of Synaptophysin, a pre-synaptic marker and post-synaptic density protein-95 (PSD-95, also known as Disks large homolog 4 (DLG4)). PSD-95, a member of the PDZ scaffolding protein family, is a well-established post-synaptic protein that stabilizes young synapses and anchors other synaptic proteins to post-synaptic densities [31,32]. Synaptophysin is a pre-synaptic vesicle protein present in the neurites during brain development and regulates synaptic vesicle cycling [33,34]. miR-26b-5p was overexpressed in NSCs and the cells were allowed to differentiate for 72h. Our results showed that miR-26b-5p overexpression resulted in a significant increase in Synaptophysin and a significant decrease in PSD-95 protein expressions in differentiating cells (Figure 3A,B). In addition, immunofluorescence analysis confirmed a moderate increase in Synaptophysin protein (Figure 3C) and the decrease in PSD-95 (Figure 3D) in differentiating cells following miR-26b-5p overexpression.

In order to verify that miR-26b-5p alters the expression of synaptic proteins via Mecp2, siRNA-mediated knockdown of Mecp2 in NSCs from embryos of control mice were performed and the expression of synaptic proteins, PSD-95 and Synaptophysin were analyzed upon differentiation of NSCs. The knockdown efficiency of Mecp2 in NSCs was found to be nearly 40%. Knockdown of Mecp2 in NSCs resulted in significant upregulation of Synaptophysin protein and significant downregulation of PSD-95 following differentiation (Figure 3E,F), which correlated with the results obtained following miR-26b-5p overexpression. Moreover, immunofluorescence analysis of differentiating cells revealed an increase in Synaptophysin protein (Figure 3G) following Mecp2 knockdown when compared to negative control.

### 3.5. miR-26b-5p Enhances Neurite Formation via Mecp2 in NSCs 

**Figure 4:**
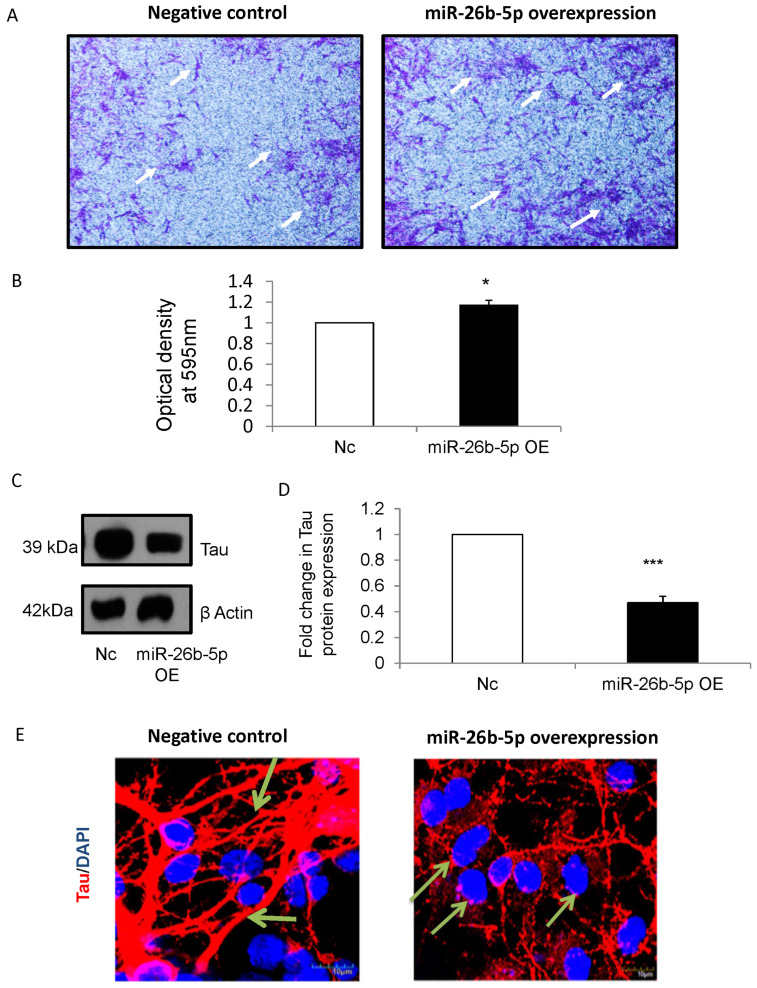

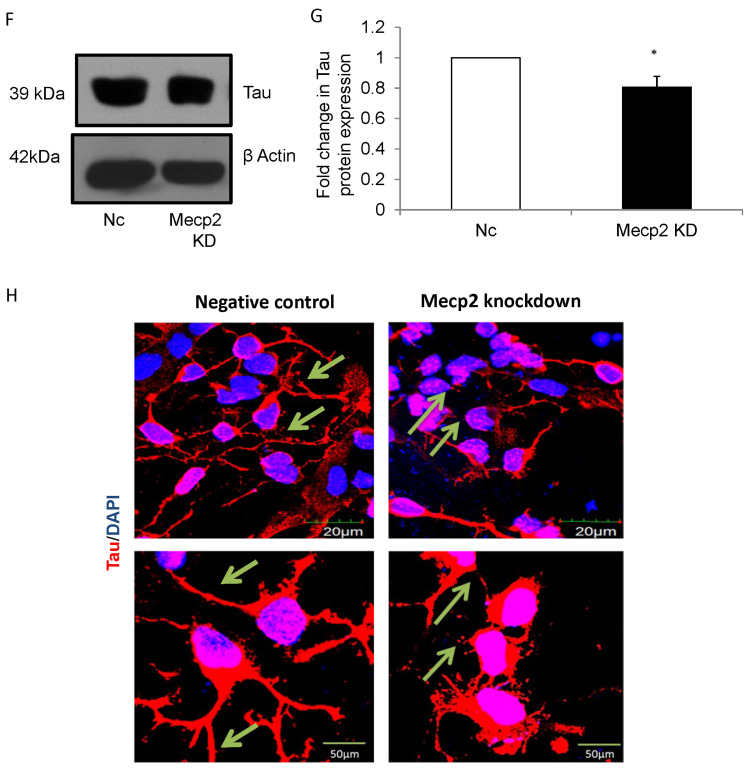
(**A**) Microscopic images showing increased number of neurites in NSCs overexpressed with miR-26b-5p (right panel) following differentiation, when compared to that of negative control (left panel). (**B**) The optical density was measured at absorbance 595 nm following extraction of the stain using stain extraction solution. There was a significant increase in optical density in NSCs following miR-26b-5p overexpression (closed bar) when compared to that of negative control (open bar). (**C**) Representative blots showing the expression of Tau protein in NSCs following miR-26b-5p overexpression. β actin was used as an internal control. There was a marked downregulation in Tau protein in NSCs following miR-26b-5p overexpression. (**D**) Densitometry analysis shows significant downregulation of Tau protein (closed bar) in NSCs following miR-26b-5p overexpression, when compared to that of negative control (open bar). Nc—Negative control, miR-26b-5p OE—miR-26b-5p overexpression. n = 4, *** *p* < 0.001. (**E**) Confocal images showing decrease in Tau-positive axonal projections in NSCs following miR-26b-5p overexpression ((**right panels**), arrows) when compared to that of negative control (**left panels**). Nuclei are stained with DAPI. Nc—Negative control, miR-26b-5p OE—miR-26b-5p overexpression. n = 3, * *p* < 0.05. Scale bar 10 μm. (**F**) Representative blots showing expression of Tau protein in NSCs following Mecp2 knockdown. β actin was used as an internal control. (**G**) Densitometry analysis shows significant decrease in Tau expression in NSCs following Mecp2 knockdown (closed bar) when compared to that of negative control (open bar). Nc—Negative control, Mecp2 KD—Mecp2 knockdown. n = 4, * *p* < 0.05. (**H**) Confocal images showing decrease in Tau-positive axonal projections (arrows) in differentiated cells following Mecp2 knockdown (**right panel**) when compared to negative control (**left panels**). Nuclei are stained with DAPI. Scale bar: upper panels, 20 μm; lower panels, 50 μm.

During brain development, neuritogenesis (formation of neuronal processes, such as axon and dendrites) is an important step as it forms the basis for synaptogenesis [35]. In view of this, a neurite outgrowth assay was performed in which NSCs overexpressed with miR-26b-5p were seeded on cell culture inserts and allowed to differentiate for 48 h. Our results showed significantly increased neurite outgrowth in differentiating cells overexpressed with miR-26b-5p when compared to that of negative control (Figure 4A,B). 

During neurite outgrowth, there is an increase in the expression of Tau protein, which determines the polarity of neurons [36]. Since Tau protein decides the fate of neurites (to become an axon or a dendrite) and neurites are important components of synaptogenesis, the expression of Tau in differentiating cells following miR-26b-5p overexpression and Mecp2 knockdown was analyzed. There was a significant decrease in the expression of Tau protein upon differentiation (Figure 4C,D) in miR-26b-5p overexpressed NSCs when compared to negative control. Furthermore, the confocal immunofluorescence images revealed a decrease in Tau-positive axonal projections (Figure 4E, right panels) in differentiated cells following miR-26b-5p overexpression when compared to that of negative control (Figure 4E–left panels).

Similarly, there was a significant downregulation of Tau protein in differentiating cells following Mecp2 knockdown (Figure 4F,G). In addition, immunofluorescence analysis of NSCs revealed the decrease in Tau-positive axonal projections (Figure 4H, right panels) in differentiated cells following Mecp2 knockdown when compared to negative control (Figure 4H, left panels). Taken together, the findings suggest that miR-26b mediates the process of neurite outgrowth and tau expression by regulating the expression of its target Mecp2. 

### 3.6. miR-26b-5p Deregulates Synaptic Vesicle Cycling in NSCs

**Figure 5:**
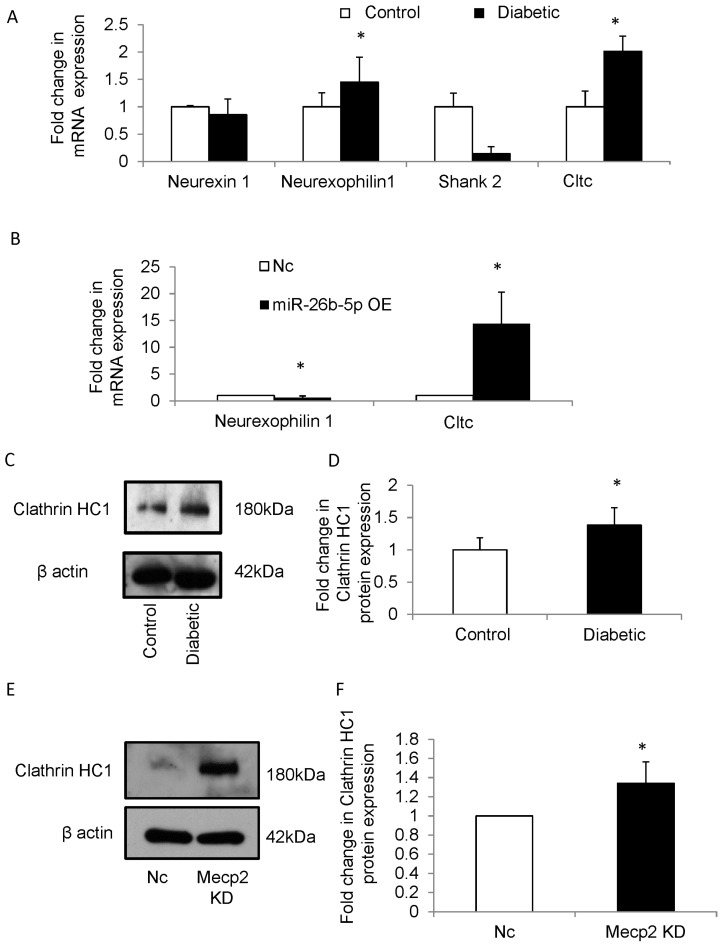
(**A**) The mRNA expression level of Neurexin1, Neurexophilin1, Cltc and Shank2 were quantified in NSCs from embryos of control and diabetic pregnancy. Out of the four genes analyzed, Neurexophilin1 and Cltc genes showed a significant increase in NSCs from diabetic pregnancy (closed bars) when compared to that of control pregnancy (open bars). n = 4, * *p* < 0.05. (**B**) NSCs were transfected with miR-26b-5p mimics and negative control probes and the expression levels of Neurexophilin1 and Cltc were analyzed using qRT-PCR. The mRNA expression of Cltc showed a significant increase in NSCs following miR-26b-5p overexpression (closed bar) when compared to negative control (open bar). Nc—Negative control, miR-26b-5p OE—miR-26b-5p overexpression. n = 4, * *p* < 0.05. (**C**) Representative blot showing expression of CLH1 protein in NSCs from embryos of control and diabetic pregnancy. There is an increase in expression of CLH1 protein in NSCs from diabetic pregnancy when compared to that of control. β actin was used as an internal control. (**D**) Densitometry quantification revealed that CLH1 expression is significantly increased in NSCs from diabetic pregnancy (closed bar) when compared to control (open bar). C-Control, D-Diabetic. n = 3, * *p* < 0.05. (**E**) Representative blot showing expression of CLH1 protein in NSCs following Mecp2 knockdown. β actin was used as an internal control. There is an increase in CLH1 expression in NSCs following Mecp2 knockdown when compared to that of negative control. β actin was used as an internal control. (**F**) Densitometry analysis shows significant increase in CLH1 protein expression in NSCs following Mecp2 knockdown (closed bar) when compared to that of negative control (open bar). Nc—Negative control, Mecp2 KD—Mecp2 knockdown. n = 4, * *p* < 0.05.

Next, we examined the expression of several synaptic proteins that are predicted targets of miR-26b-5p. Using the TargetScan (mouse) software, Shank2, Clathrin HC-1, Neurexophilin1 and Neurexin1 were predicted as targets of miR-26b-5p (Supplementary Figure S1B). qRT-PCR was performed to analyze the expression of the synaptic genes in NSCs from embryos of control and diabetic mice (primers are listed in Table 1). The mRNA expression levels of Neurexophilin1 (*Nxph1*) and Clathrin HC-1 (*Cltc*) were significantly upregulated in NSCs from embryos of diabetic pregnancy when compared to controls (Figure 5A). Furthermore, the mRNA expression of *Nxph1* and *Cltc* were analyzed in NSCs following overexpression of miR-26b-5p. *Cltc* gene was found to be significantly upregulated (nearly 15 folds), while *Nxph1* was downregulated in NSCs following miR-26b-5p overexpression when compared with negative control (Figure 5B). 

The *Cltc* gene encodes the Clathrin Heavy Chain 1 (CLH1) protein, which forms the major part of coating pits and vesicles involved in receptor-mediated endocytosis and is important for various neurodevelopmental processes such as cell fate specification, determining neuronal polarity, migration, axonal guidance and outgrowth [37]. Since *Cltc* gene was observed to be significantly upregulated in NSCs from embryos of diabetic pregnancy, the expression of the encoded protein CLH1 was analyzed. Western blot analysis revealed significant upregulation of CLH1 in NSCs from diabetic pregnancy (Figure 5C,D).

Furthermore, recent proteomic studies suggest dysregulated Clathrin-mediated endocytosis in Mecp2 mutant models [38]. Since it is possible that upregulation of CLH1 in NSCs of embryos from diabetic pregnancy and in NSC following miR-26b-5p overexpression is mediated via Mecp2, we examined the expression of CLH1 following mecp2 knockdown. We observed that the expression of CLH1 was upregulated significantly in NSCs following Mecp2 knockdown (Figure 5E,F), indicating that miR-26b-5p may regulate CLH1 via Mecp2.

## 4. Discussion

The maternal environment influences normal brain development and function during embryogenesis [39]. Metabolic alterations such as hyperglycemia during pregnancy have been found to result in a spectrum of congenital neurological defects, including structural and long-term functional defects [40], which determine the health and behavior of the offspring. Though early diagnosis and appropriate management of diabetes during pregnancy have reduced the incidence of structural defects such as neural tube defects (NTDs) in humans to a greater extent, infants of diabetic mothers are at risk of neurofunctional deficits later in life [41]. A recent meta-analysis showed a significant association between maternal diabetes and the risk of neurodevelopmental disorders, including autism in offspring [1,2,42]. Therefore, it is important to identify and understand the molecular mechanisms underlying maternal diabetes-induced changes in brain development and function.

The normal development of the brain begins with neurulation, and diverse processes such as proliferation, migration, differentiation, synaptogenesis, synaptic pruning and myelination are critical determinants of brain function since they mediate precise wiring in the brain [43,44]. These processes have been shown to be regulated by several molecules, including Mecp2. Altered Mecp2 expression has been implicated in many neurodevelopmental disorders such as Rett syndrome, mental retardation, epilepsy and Angelman syndrome [45]. Specifically, loss of Mecp2 is associated with defective synaptogenesis [45]. Mecp2 is a highly complex protein and its transcript generates two protein isoforms, namely Mecp2-e1 and e2 that are 498 and 486 amino acids long in humans (501 and 484 amino acids long in mice) and have differences in the N-terminal [46]. The longer isoform (i.e., Mecp-e1) has been shown to be the predominant isoform in the brain of humans and mice [47]. Mecp2 is known to be regulated by microRNAs and DNA methylation, as well as post-translational modifications such as phosphorylation, sumoylation and acetylation. To date, at least seven microRNAs (miR-7b, miR-22, miR-124a, miR-132, miR-212, miR-483-5p, and miR-511) [16] have been found to target and regulate the expression of Mecp2 in the brain. However, it is predicted that Mecp2 has complementarity to several more miRNAs as it has a large 3’UTR [48]. 

In our study, we used NSCs differentiated for 72h to examine the early changes in neurite formation and expression of synaptic markers. It has been shown that rat NSCs can express low levels of pre-synaptic and post-synaptic genes. Further these studies showed that, on Day 5 post-differentiation, the expression of synaptic proteins such as Synaptophysin was localized to the perinuclear region (similar to our immunostaining results), suggesting that immature differentiating neurons also express synaptic proteins [49]. Moreover, both Synaptophysin and PSD-95 are known to be associated with Mecp2, and the expression patterns of these proteins have been shown to be Mecp2 dependent [30,50,51]. Synaptophysin is an early synaptic vesicle protein that is found in abundance in both inhibitory and excitatory synapses during synaptogenesis, and mediates synaptic stability [33]. In humans, Synaptophysin expression has been reported in developing brains since mid-gestation until early adulthood [51,52]. Furthermore, Synaptophysin also has diverse roles such as calcium binding, channel formation, exocytosis and recycling of synaptic vesicles by endocytosis [52], and an increase in Synaptophysin expression is attributed to an increase in synaptic activity [53,54]. PSD-95 is a membrane protein that is a potent scaffolder during development and aids in maintaining the structure and strength of the post-synaptic densities in excitatory synapses [55,56]. PSD-95 is localized to dendritic spines, an important component of synapses, and found to alter their morphology and stability [57]. Deregulation of PSD-95 expression is associated with neurological disorders, including schizophrenia and Fragile X syndrome [58,59,60]. Moreover, PSD-95 is essential for the maturation and stabilization of excitatory synapses [61]. In our study, the expression of PSD-95 was downregulated, while Synaptophysin was upregulated in NSCs following miR-26b-5p overexpression or Mecp2 knockdown, suggesting that Mecp2/miR-26b-5p may regulate the maturation of synapses [62]. 

Neurites are vital components in synaptogenesis as they mature into either axons or dendrites [63]. Since axons and dendrites are inevitable components in a synapse, assessing the ability of differentiating NSCs to form neurites following miR-26b-5p overexpression is deemed necessary to understand the role of miR-26b-5p in synaptogenesis. The significant increase in neurite number (as observed by optical density value) in NSCs overexpressed with miR-26b-5p, when compared to negative control, emphasizes the role of miR-26b-5p in inducing neurite formation. Primarily, the stabilization of Tau protein is essential for a neurite to mature into an axon or dendrite. Tau determines the polarity of NSCs as the neurite that expresses Tau becomes the axon while the other neurites become dendrites [36]. Furthermore, Mecp2 has also been shown to regulate the expression of Tau [64]. In the present study, the expression of Tau protein was found to be downregulated in NSCs following miR-26b-5p overexpression, and in NSCs following Mecp2 knockdown. Thus, the reduced Tau expression impedes the polarization of differentiating NSCs, i.e., the axonal and dendritic compartments are not well defined, and hence the neurites fail to attain maturity. Taken together, the inverse expression pattern between Synaptophysin and PSD-95 proteins, downregulated Tau expression and the presence of increased immature neurites suggest the presence of increased immature synapses in NSCs following miR-26b-5p overexpression. 

Among the putative targets of miRNA-26b, *Cltc* showed a stark increase in NSCs following miR-26b-5p overexpression (~15-fold increase). Generally, living cells internalize molecules from their environment using a process called endocytosis. Endocytosis can occur by five mechanisms of which Clathrin-mediated endocytosis is a classical model of internalization [65]. During embryogenesis, the prime function of Clathrin-dependent machinery in the embryonic cells is to deliver the extracellular cues that are essential for proper synaptic formation, axonal outgrowth and dendritic maintenance [66]. Synapses are enriched with Clathrin, a predominant vesicular coat protein, and the synaptic vesicle cycling is pivotal for the normal function of a synapse [67]. An increase in Clathrin-coated vesicles (CCV) and Clathrin expression was observed in the brains of Auxillin (a co-chaperone protein required for Clathrin uncoating) knockout mice, in which the process of Clathrin-dependent endocytosis was impaired, leading to postnatal mortality [68]. In the present study, CLH1 protein expression was observed to be increased in NSCs from embryos of diabetic pregnancy and in NSCs following miR-26b overexpression. Further, upregulation of CLH1 in NSCs following Mecp2 knockdown indicates that there is impairment in the Clathrin-dependent endocytic machinery in differentiated NSCs, which may lead to stagnation of CCVs in the synapse and thus disrupt synaptogenesis [68]. So far there is no direct evidence showing Mecp2-mediated regulation of Clathrin. Recent proteomic studies suggest that Clathrin-mediated endocytosis is dysregulated in Mecp2 mutant Rett syndrome models [38]. The current results suggest a potential interaction between Mecp2 and Clathrin, since CLH1 was upregulated upon knockdown of Mecp2 as well as in NSCs from diabetic pregnancy (in which Mecp2 expression was downregulated). However, further studies are required to validate the interaction between Mecp2 and CLH1. Overall, maternal diabetes appears to disrupt Mecp2-mediated synaptic plasticity, and Clathrin-mediated endocytosis that is required for recycling synaptic vesicles [69]. 

Several studies have demonstrated that altered synaptogenesis and synaptic functions underlie several neurological diseases (reviewed in [70,71]) since proper synaptic communication is required for precise brain function. From the results obtained, it appears that maternal diabetes perturbs synaptogenesis and synaptic functions in differentiated cells via miR26b and its target Mecp2, which may contribute to neurodevelopmental disorders in offspring. 

## 5. Conclusions

**Figure 6:**
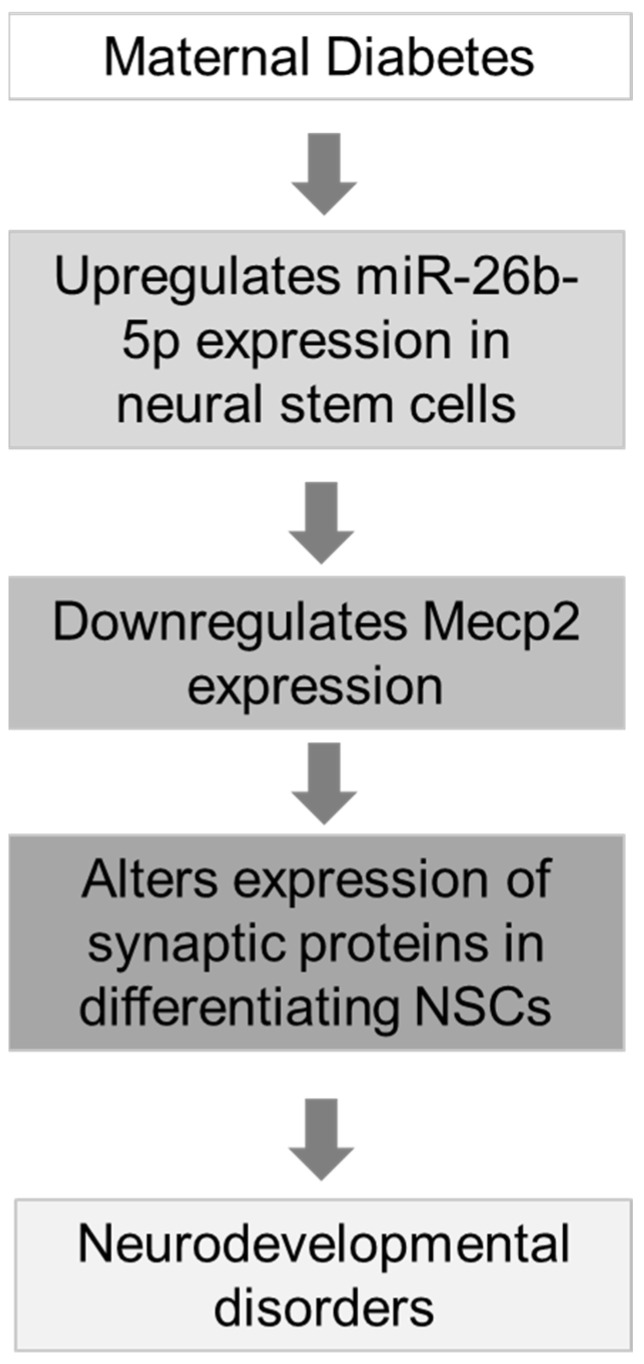
Overall summary. Maternal diabetes increased the expression of miR-26b-5p, which targets Mecp2 in NSCs, resulting in defective synaptogenesis and thus leading to neurodevelopmental disorders.

In this study, we showed that Mecp2 expression is epigenetically regulated during brain development via miR-26b, and that deregulation of miR-26b-Mecp2 in NSCs and synaptogenesis in differentiating neurons derived from embryos of diabetic pregnancy may underlie neurodevelopmental disorders associated with maternal diabetes (Figure 6). 

## Figures and Tables

**Table 1:** List of qPCR primers.

Gene	Left Primer	Right Primer
Mecp2	GGAAGGGACTGAAGACCTGC	TGGTGGTGATGATGGTGCTC
β Actin	GAAGAGCTATGAGCTGCCTGA	GGATTCCATACCCAAGAAGGA
Cltc	GCTCCAGAACCTGGGTATCA	CAGGATTCATGATGGCACTG
Shank2	TTCCCCCACCACATACAACT	GTCCAGGGAAAATCTGTCCA
Neurexin1	AGGACATTGACCCCTGTGAG	TGGCATAGAGGAGGATGAGG
Neurexophilin1	AAGTCACGTGTCCTGGCTCT	TTAACTCCGCAGGCTTCAGT

**Table 2:** List of miRNA primers and their sequence.

miRNA Primers	Sequence
U6(hsa/rna/mus)	CACGAATTTGCGTGTCATCCTT
hsa-miR-26b	UUCAAGUAAUUCAGGAUAGGU
hsa-miR-26a	UUCAAGUAAUCCAGGAUAGGCU

**Table 3:** List of SMARTpool probes used in the study.

Gene	SMARTpool Probes	Sequence
Mecp2	D-044034-01	CGAGGAGGCUCACUGGAAA
	D-044034-02	ACACGAAAGCUUAAACAAA
	D-044034-03	GGACUGAAGACCUGCAAGA
	D-044034-04	CAGCUAAGACUCAGCCUAU
siGENOME	Non-targeting siRNA pool #1	UAGCGACUAAACACAUCAA UAAGGCUAUGAAGAGAUAC AUGUAUUGGCCUGUAUUAG AUGAACGUGAAUUGCUCAA

**Table 4:** List of miRNA mimics and inhibitors.

**Catalog No.**	**miRNA Mimics**	**Sequence**
MC12899	hsa-miR-26b-5p	UUCAAGUAAUUCAGGAUAGGU
4464058	Negative control	TAACACGTCTATACGCCCA
**Catalog No.**	**miRNA inhibitors**	**Sequence**
199006-001	Negative control	TAACACGTCTATACGCCCA
4104759-001	hsa-miR-26b-5p	ACCTATCCTGAATTACTTGA

## Data Availability

Not applicable.

## Supplementary Materials

The following supporting information can be downloaded at: https://www.mdpi.com/article/10.3390/cells12111516/s1, Figure S1. Bioinformatic prediction showing that (A) miR-26 family targets Mecp2 and (B) miR-26b-5p targets synaptic genes.
