# Supplementary material for: Maternal Diabetes Deregulates the Expression of Mecp2 via miR-26b-5p in Mouse Embryonic Neural Stem Cells
Source: Cells. 2023 May 30;12(11):1516. doi: 10.3390/cells12111516 (PMC10252249; doi:10.3390/cells12111516)
Supplement: Supplementary file 1 [file cells-12-01516-s001.zip › Supplementary Figure S1.pdf]

(A) Bioinformatic analysis predicts that miR-26 family targets Mecp2 that is involved in Synaptogenesis

<table>
<tr><th></th><th>Predicted consequential pairing of target region (top) and miRNA (bottom)</th></tr>
<tr><td>Position 4359-4365 of MECP2 3' UTR
mmu-miR-26b-5p</td><td>5' ...AUGCUAAAGGGGAAAACUUGAAC...
||||||
3' UGGAUAGGACUUAAUGAACUU</td></tr>
<tr><td>Position 4359-4365 of MECP2 3' UTR
mmu-miR-26a-5p</td><td>5' ...AUGCUAAAGGGGAAAACUUGAAC...
||||||
3' UCGGAUAGGACCUAAUGAACUU</td></tr>
</table>

(B) Bioinformatic analysis predicts other synaptic proteins that are putative targets of miR-26b-5p

<table>
<tr><th></th><th>Predicted consequential pairing of target region (top) and miRNA (bottom)</th></tr>
<tr><td>Position 1187-1193 of Cltc 3' UTR
mmu-miR-26b-5p</td><td>5' ...AUGAGUGUUACACUGACUUGAAA...
||||||
3' UGGAUAGGACUUAAUGAACUU</td></tr>
<tr><td>Position 4159-4166 of Shank2 3' UTR
mmu-miR-26b-5p</td><td>5' ...GGCCGACCUUCUCUGUACUUGAA...
||| |||||||
3' UGGAUAGGACUUA--AUGAACUU</td></tr>
<tr><td>Position 2744-2750 of Nrxn1 3' UTR
mmu-miR-26b-5p</td><td>5' ...UUUUGAAGGAAAAAGACUUGAAU...
||||||
3' UGGAUAGGACUUAAUGAACUU</td></tr>
<tr><td>Position 12506-12512 of Nxph1 3' UTR
mmu-miR-26b-5p</td><td>5' ...GAAGGGGCUUGUAUAUACUUGAG...
|||||||
3' UGGAUAGGACUUAAUGAACUU</td></tr>
</table>

Supplementary Figure S1. Bioinformatic prediction showing that (**A**) miR-26 family targets Mecp2 and (**B**) miR-26b-5p targets synaptic genes.
